# SIMULTANEOUS CO-OCCURRENCE OF POROKERATOSIS OF MIBELLI WITH DISSEMINATED SUPERFICIAL ACTINIC POROKERATOSIS

**DOI:** 10.4103/0019-5154.57625

**Published:** 2009

**Authors:** Vandana Mehta, C Balachandran

**Affiliations:** *From the Department of Skin and STD, Kasturba Hospital, Manipal, Karnataka - 576 104, India. E-mail: vandanamht@yahoo.com*

Sir,

Porokeratosis is a group of hereditary or acquired disorders of epidermal keratinization, characterized by keratotic lesions with an atrophic center and a prominent peripheral ridge, with a typical histologic hallmark, the cornoid lamella. Since its first description by Mibelli and Respighi in 1893, a bewildering number of porokeratosis types and morphological forms have been reported. Classically, five clinical variants are recognized: classic porokeratosis of Mibelli (CPM), disseminated superficial actinic porokeratosis (DSAP), disseminated superficial porokeratosis (DSP), porokeratosis palmaris et plantaris disseminata, and linear porokeratosis. Apart from these five clinical variants, a number of atypical morphological forms such as facial porokeratosis, giant porokeratosis, punched-out porokeratosis, hypertrophic verrucous porokeratosis and reticulate porokeratosis also have been reported in literature.[1] We report a case with simultaneous presence of DSAP and CPM in a young lady with familial occurrence of the same.

A 43-year-old lady presented with a pruritic hyperpigmented annular keratotic plaque with raised thready borders on the flexor aspect of the right forearm since childhood [Figure 1]. Similarly, such annular pigmented lesions appeared over the years on the face, neck, back and extensor forearms associated with photosensitivity [Figure 2]. Her past medical history was noncontributory; however, she gave a history of similar lesions in her mother and sister. Histopathological examination of a representative lesion on the face and forearm revealed features typical of porokeratosis [Figures 3 and 4].

**Figure 1:**
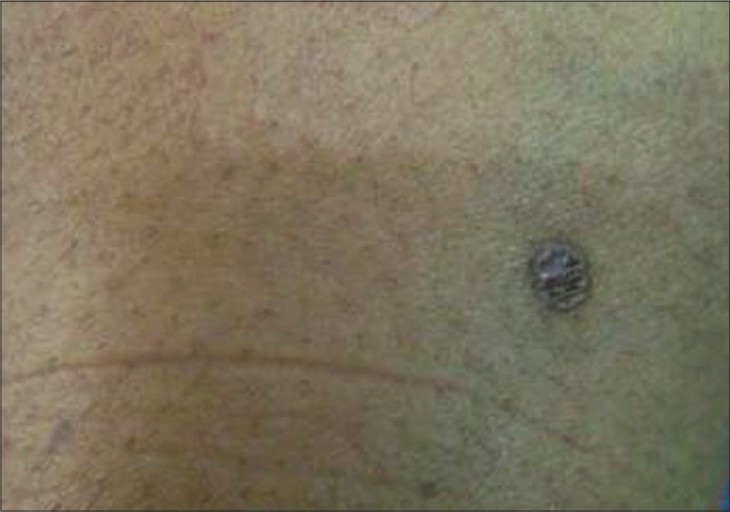
Close up of an annular plaque on the forearm with raised thready borders

**Figure 2:**
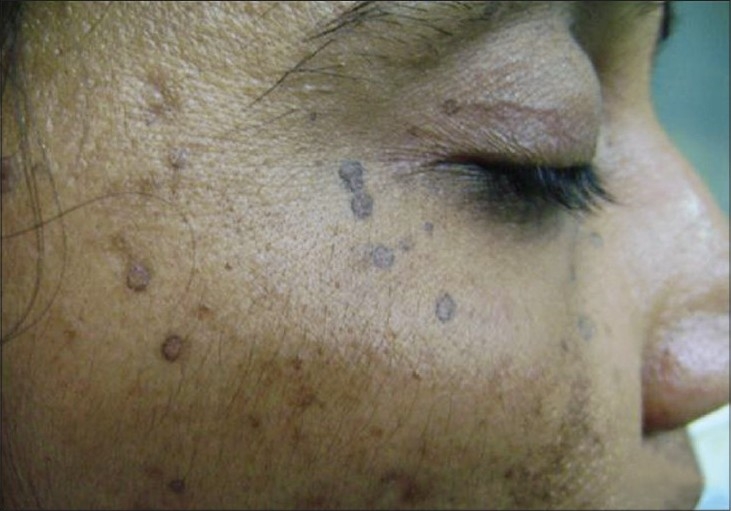
Multiple annular pigmented plaques on the face

**Figure 3:**
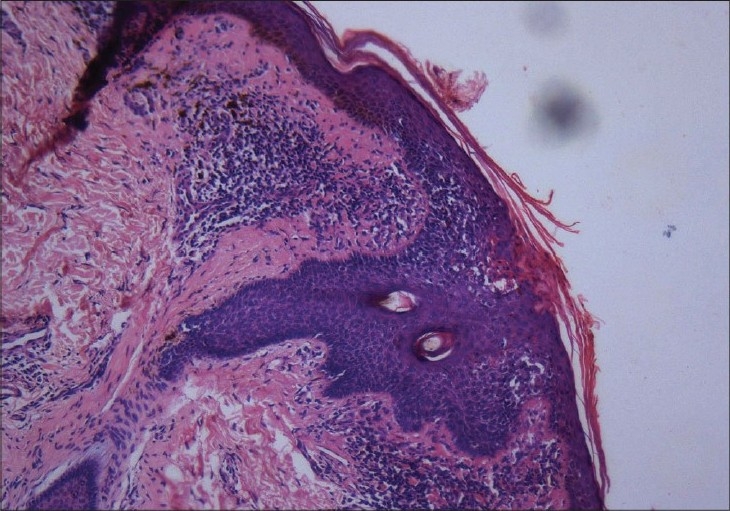
10× photomicrograph showing the cornoid lamella with inflammatory infiltrate in the dermis

**Figure 4:**
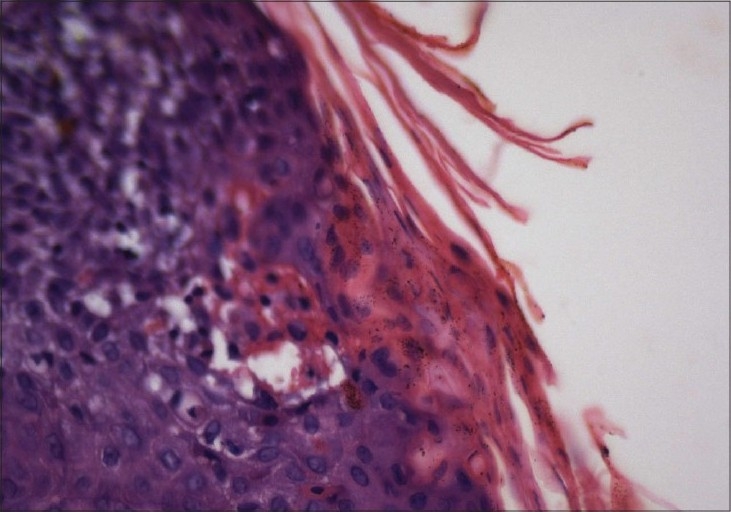
40× photomicrograph showing the close up of the cornoid lamella

Porokeratosis (PK) are inherited as an autosomal dominant trait; however, sporadic cases are also known to occur. DSAP is the most common of the five clinical variants of PK and usually develops in the third and fourth decades of life, with a female preponderance. It is characterized by multiple, brown, annular, keratotic lesions predominantly on the sun-exposed areas. Facial lesions are seen in approximately 15% of patients and lesions spare the axillary vaults, inguinal folds, perigenital regions, palms, soles and mucous membranes.[1] Chenosky *et al*. first described DSAP in 1969 in Texas population.[2] There is good evidence that ultraviolet light can precipitate the development of new lesions or exacerbate preexisting DSAP, and this observation is consistent with its common clinical presentation in the sun-exposed areas. Unlike other forms of PK, DSAP does not appear to have significant risk of malignant change. Genetic studies have mapped the loci responsible for DSAP to chromosomes 12q, 15q, and 18q. Recently, several mutations have been identified in the SSH1 gene on chromosome 12, which encodes a phosphatase that plays a pivotal role in actin dynamics.[3] The exact pathogenesis of porokeratosis is not clear;however, it has been assumed that a focal expanding clone of abnormal cells gives rise to the coronoid lamella.[4] Rarely, concurrence of different variants of PK has been observed. The coexistence of different variants in one patient or in several members of an affected family indicates different phenotypic expressions of a common genetic entity which could possibly be explained due to simultaneous expression of closely linked genes.[5]

Our case had lesions that were typical of DSAP on the face, neck, back and porokeratosis of Mibelli on the right forearm. Interestingly, she also gave history of a familial occurrence of such lesions in her mother and sister, which is quite rare.[6] Reported cases of mixed PK in literature mostly involve DSAP and linear PK. Only three cases of Porokeratosis of Mibelli have been described in association with DSAP.[5] The rarity of this association along with a familial occurrence prompted us to report this case. Our patient was commenced on topical retinoic acid along with sun-protective measures following which she was lost to follow up.

## References

[1] Palleschi GM, Torchia D (2008). Porokeratosis of Mibelli and superficial disseminated porokeratosis. J Cutan Pathol.

[2] Anderson DE, Chernosky ME (1969). Disseminated superficial actinic porokeratosis: Genetic aspects. Arch Dermatol.

[3] Xia JH, Yang YF, Deng H, Tang BS, Tang DS, He YG (2000). Identification of a locus for disseminated superficial actinic porokeratosis at chromosome 12q 23.2-24.1. J Invest Dermatol.

[4] Reed RJ, Leone P (1970). Porokeratosis: A clonal keratosis of the epidermis. Arch Dermatol.

[5] Dover JS, Phillips TJ, Burns DA, Krafchik BR (1986). Disseminated superficial actinic porokeratosis, coexistence with other porokeratotic variants. Arch Dermatol.

[6] Seghal VN, Dube B (1973). Porokeratosis of Mibelli in a family. Dermatologica.

